# Reply to “Dyslexia: Still Not a Neurodevelopmental Disorder”

**DOI:** 10.3390/brainsci9030061

**Published:** 2019-03-14

**Authors:** Gorka Fraga González, Iliana I. Karipidis, Jurgen Tijms

**Affiliations:** 1 Department of Developmental Psychology, University of Amsterdam, 15916 1001 NK Amsterdam, The Netherlands; jurgentijms@gmail.com; 2 Rudolf Berlin Center, 44401 Amsterdam, The Netherlands; 3 Department of Child and Adolescent Psychiatry and Psychotherapy, Psychiatric Hospital, University of Zurich, 8032 Zurich, Switzerland; iliana.karipidis@kjpd.uzh.ch; 4 Neuroscience Center Zurich, University of Zurich and ETH Zurich, 8057 Zurich, Switzerland; 5 RID, 1018 WS Amsterdam, The Netherlands

**Keywords:** dyslexia, reading difficulty, neurodevelopmental disorder, neuroimaging

## Abstract

In a recent opinion article, we explained why we think that defining developmental dyslexia as a neurodevelopmental disorder and neuroimaging studies on dyslexia are useful. A recent response has made some claims of generalized misinterpretation and misconception in the field. Since that was a direct reply to our article, we would like to clarify our opinion on some of those claims.

## Reply

First of all, we would like to clarify that the main goal of the opinion article [1] (henceforth FKT18) was not to rebuke the points of view expressed in [2], although this was the case in some instances. The goal of our article was to share why we think defining developmental dyslexia as a neurodevelopmental disorder and neuroimaging studies on dyslexia are useful. We believe this was expressed clearly in the paper. In view of the reply from the authors in [3] (henceforth [PP19]) and their explicit and strong claims about our field, we feel compelled to respond. In FKT18 we argued why dyslexia fits comfortably within the general criteria for mental disorder and more specifically for neurodevelopmental disorder. In contrast to the title of their paper, [PP19] do not seem to provide counterarguments against dyslexia as a neurodevelopmental disorder, in particular, but question the validity of mental disorders, in general, and especially question the role of neuroscience in clinical (and educational) research. To avoid a repetition of moves, we will focus on this last issue in our response. 

The first point we would like to comment on is the apparent dismissal of current research using neuroimaging to study learning disabilities. We believe the authors do not sufficiently justify their views expressed in statements such as “sloppy thinking and misinterpretation of data that is rampant in the field”. We also think this claim portrays a distorted picture of neuroimaging research in the field of learning disabilities, which could easily be generalized to the study of any other psychiatric disorder using neuroimaging. We find that the arguments offered by [PP19] to substantiate this strong opinion are too unspecific and occasionally misleading. For instance, the reference to the use of statistical thresholds to determine what we call a pattern or what we consider worthy of further interpretation. This is an issue that extends beyond neuroimaging research, and [PP19] do not discuss what method of inquiry would be acceptable to study individuals within a population without resorting to thresholds and define, for instance, who qualifies as a poor reader and requires more attention. In addition, in their discussion on the misinterpretation of neuroimaging data, the authors imply that most researchers are either not aware of the major limitations to the methods or make misleading claims about the findings. We are of the opinion that, more often than not, caution is exerted in neuroimaging reports to avoid overinterpretation. To imply otherwise and suggest malpractice in the field would require some evidence, which we find lacking in [PP19]’s response. Meta-analysis and reviews have pointed at the lack of convergence of some reports, and in-depth reviews have raised issues like replicability or interpretational caveats on MRI research. However, this does not equate to widespread misinterpretation of results in the community or invalidate the notion of a neurodevelopmental disorder. Importantly, [PP19] do not clarify what analysis approach or what type of findings would, according to their view, qualify for the legitimate interpretation of neuroimaging data. Instead, their description of MRI results as observing “colored blobs here and there” appears to be more focused on creating a sense of “sloppy thinking” in the field rather than on discussing the interpretational caveats of whole-brain MRI analysis. Again, we think the discussion lacks specificity and completeness (limited to a particular analytic approach and technique) to conclude that there is a “standard practice of misinterpretation” and that “every proclamation that an atypical activation pattern has been demonstrated is necessarily incorrect and misleading”. 

Having said that, we also would like to address some specific misconceptions concerning [PP19]’s critique to our paper. First, [PP19] note that, to establish abnormality, deviations have to be shown before the onset of skill acquisition, something they claim our paper failed to show. It is important to note that some deviations will only be present at the moment a network subserving skill performance is forming. In the case of dyslexia, areas for auditory/spoken language processing and visual processing need to possess a flexibility to adapt to the requirements of the learned skill and thus to converge into an integrated neural network for processing written language—a development that might expose abnormalities that were not detectable before. Nonetheless, we cited several studies showing deviations early in development, e.g., in auditory processes in infants that predict individual reading levels 14 years later (e.g., [4]). Additionally, both Karapidis et al. [5,6] at a neurophysiological level and Horbach et al. [7] at a behavioral level, as we noted in FKT18, chose to mimic this network development by learning artificial letter–speech sound associations in preliterate children. These studies showed that failure in this differentiated at-risk from non-risk children and was a predictor for reading problems on an individual level years later. Second, [PP19]’s emphasis on MRI seems to suggest that this is the only technique used in the field of reading disabilities, which was referred to in FKT18. It is therefore good to note that, as visual word recognition and the related letter–speech sound integration processes evolve in a very short temporal window, a large part of the evidence is electroencephalogram (EEG)-based, as reported in FKT18. Importantly, both within (f)MRI-based techniques as well as between (f)MRI and other neurophysiological techniques, there is a high convergence in the findings on neurocognitive underpinnings of both typical reading processes and failures thereof in dyslexic readers (see [8] for an overview). Another misconception we would like to correct is that the evidence on dyslexia is not only based on between-group comparisons but also on within-group associations between neurocognitive findings and reading problems (e.g., [9,10], see also [11] demonstrating on a cognitive level the presence of phonologically-based deficits in the large majority of individuals with a reading disability). 

Additionally, [PP19] state that it is absurd to believe that findings from the field of (cognitive) neuroscience can play an instrumental role in developing reading interventions, and they wonder what the role of the neurologist in treating a child’s reading disability would be. This seems to be a misunderstanding; what we meant by instrumental is that insight into the neural mechanisms of learning to read and the failure thereof can be translated to optimize the (behavioral) intervention for reading disabilities. For a more extensive overview on the role of (cognitive) neuroscience in educational or clinical practice, see for instance [12,13].

Ultimately, it seems that the main point of divergence between our views has to do with what we consider a neurodevelopmental disorder to be. This is not a trivial question and would require a more philosophical treatment (see [14] for a well-argued opinion paper). In our article, a rather pragmatic view of disorders underlies our opinion in favor of *using* the label “neurodevelopmental disorder” to refer to dyslexia. This view does not imply a qualitative judgment of whether brain differences are “wrong” or “bad”. The aim is to identify aspects of brain function and structure that lead to the development of poor reading skills or are a consequence of poor reading. We do not discuss the philosophical nature or the “reality” of a disorder as this falls out of our scope. [PP19] argue why dyslexia does not qualify as a neurodevelopmental disorder and focused on the topic—dyslexia *is* not a neurodevelopmental disorder. Their use of expressions such as “frank neurodevelopmental failure” or “wrong” imply a certain underlying view on the nature of the disorder that, to us, seems to be incompatible with the nature of potential atypical pathways in neurocognitive development. Unfortunately, we find no clear argument for what exactly, according to [PP19], would qualify as a disorder, rather just “specific things in specific brains which have gone wrong in specific ways” [PP19]. 

Finally, we thank [PP19] for their interest in our opinion and for acknowledging many of the points of convergence in both views. The interest on promoting optimal reading skills among populations seems to be a clear one.
